# Transcriptomic Profiling of Pleural Effusions: Differences in Malignant and Infectious Fluids

**DOI:** 10.3390/medicina60030424

**Published:** 2024-03-01

**Authors:** Lucía Zamora-Molina, Eduardo García-Pachón, Marta Amorós, Julia Gijón-Martínez, Judith Sánchez-Almendro, Carlos Baeza-Martínez, Luis Hernández-Blasco, Antonio Galiana

**Affiliations:** 1 Section of Respiratory Medicine, Hospital General Universitario de Elche, 03203 Elche, Alicante, Spain; egpachon@gmail.com (E.G.-P.); baezamartinez.c@gmail.com (C.B.-M.); 2 Department of Clinical Medicine, Universidad Miguel Hernández de Elche, 03202 Elche, Alicante, Spain; lhernandez5562@gmail.com; 3 Department of Microbiology-FISABIO, Hospital General Universitario de Elche, 03203 Elche, Alicante, Spain; asjmarta@gmail.com (M.A.); juliagijonmartinez@gmail.com (J.G.-M.); judhsa@gmail.com (J.S.-A.); antoniogaliana1@gmail.com (A.G.)

**Keywords:** biomarkers, high-throughput sequencing, inflammation, mRNA, pleural effusion, transcriptome

## Abstract

*Background and Objectives*: Different cellular and molecular processes are involved in the production of malignant and infectious pleural effusions. However, the underlying mechanisms responsible for these differences or their consequences remain incompletely understood. The objective of this study was to identify differences in gene expression in pleural exudates of malignant and infectious aetiology and establish the possible different biological processes involved in both situations. *Materials and Methods*: RNA transcriptomic analysis was performed on 46 pleural fluid samples obtained during diagnostic thoracocenteses from 46 patients. There were 35 exudates (19 malignant and 16 infectious effusions) and 11 transudates that were used as a reference control group. Differential gene expression analysis for both exudative groups was identified. An enrichment score using the Human Kegg Orthology database was used for establishing the biological processes associated with malignant and infectious pleural effusions. *Results*: When comparing malignant exudates with infectious effusions, 27 differentially expressed genes with statistical significance were identified. Network analysis showed ten different biological processes for malignant and for infectious pleural effusions. In malignant fluids, processes related to protein synthesis and processing predominate. In infectious exudates, biological processes in connection with ATP production prevail. *Conclusions*: This study demonstrates differentially expressed genes in malignant and infectious pleural effusions, which could have important implications in the search for diagnostic or prognostic biomarkers. In addition, for the first time, biological processes involved in these two causes of pleural exudates have been described.

## 1. Introduction

Pleural effusions can occur through two different mechanisms: a noninflammatory imbalance between the hydrostatic and oncotic pressure within the capillaries (which causes a transudate effusion) and an inflammatory alteration that precipitates a pleural fluid accumulation (exudative effusion) [1,2]. Pleural effusions are very common in clinical practice. Their prevalence is estimated at about 400 cases/10,000 inhabitants [1]. Pleural exudates may be due to a variety of causes, the most frequent being infections and malignant diseases. It is often difficult to establish the cause and prognosis of these effusions [2,3]. For this reason, many studies are aimed at the discovery of new biomarkers to aid in the diagnosis of the aetiology of exudates or to aid in prognosis. Regarding infectious pleural effusions, the most frequently isolated microorganisms are aerobic Gram-positive bacteria (streptococcus, staphylococcus), anaerobes, and Gram-negative bacteria (enterobacteria, *Escherichia coli*, and *Haemophilus influenzae*), in order of frequency [4]. Regarding malignant pleural effusions, they are one of the main causes of pleural effusions, between 15 and 35%. In general, they are secondary to pleural metastases of the lung and breast; mesothelioma is the third most common cause.

The most recent technologies to explore potential biomarkers include proteomics (proteins), metabolomics (small molecules or metabolites), genomics (DNA), and transcriptomics (RNA) [3]. However, despite advances in research, underlying biological mechanisms are poorly understood, and results with clinical applications have not yet been obtained.

Transcriptomics is the branch of molecular genetics that has seen remarkably fast-paced development in recent years [4]. A transcriptome is a set of the RNA molecules transcribed from the genome in a given cell at a particular developmental stage and under certain conditions^,^ [5,6]. Determining the protein-coding RNA (messenger RNA [mRNA]), the transcriptional profile, means detecting the genes that are activated or repressed and producing a response to a certain pathological or physiological condition.

Now, it is possible to explore the entire mRNA of the pleural fluid using next-generation sequencing. This allows for the investigation of active genes and the biological functions involved in the development of malignant and infectious pleural effusions. By comparing the transcriptomic expression of inflammatory effusions with respect to noninflammatory effusions (transudates), we can establish which genes are activated in each of the processes, and with network analysis, we can explore the biological processes involved.

For this reason, we set out to analyse the transcriptome of malignant and infectious pleural effusions to recognize differentially expressed genes and to establish the pathways of inflammatory activation that define them. This may identify the different biological processes and may constitute a promising starting point for the selection of future diagnostic or prognostic biomarkers.

## 2. Materials and Methods

### 2.1. Patients and Ethics Approval

We studied 46 samples of pleural fluid collected from consecutive adult patients who underwent diagnostic thoracentesis according to standard clinical practice at the General University Hospital of Elche (HGUE, Alicante, Spain) between May 2018 and March 2020. Before participating in this study, all patients were given study details and signed a written informed consent. The research was approved by the HGUE Health Department’s Ethics Committee (ID of the ethics approval: PI 25/2018) and the principles of the revised Declaration of Helsinki were followed.

### 2.2. Specimen Collection

The aetiology of pleural effusions was established by examination of medical imaging, pleural fluid biochemistry, microbiology, cytology, and pleural biopsy according to usual clinical practice. Malignant pleural effusion was defined when tumour cells were found on cytological examination or in a pleural biopsy specimen or if patients had disseminated malignancy and there was no alternative explanation for the effusion. Infectious effusion was diagnosed when there was acute febrile illness with pulmonary infiltrate and responsiveness to antibiotic treatment. Transudative pleural effusions were diagnosed in patients with congestive heart disease or hepatic hydrothorax. Pleural fluid specimens were immediately stored at −80 °C until being used for RNA extraction, in RNAlater solution (Thermo Fisher Scientific, Waltham, MA, USA).

### 2.3. RNA Sequencing

For the extraction of total nucleic acids from pleural fluid samples preserved at −80 °C, the Tempus Spin RNA Isolation Reagent Kit (Thermo Fisher Scientific) was used according to the manufacturer’s instructions. The quality and integrity of the extracted RNA was assessed using a Nanodrop spectrophotometer (Thermo Fisher Scientific) to determine the concentration and 260/280 and 230/260 nm absorption ratio and also quantified by Quantus^TM^ to determine DNA concentration. In addition, agarose gel visualization was performed on aliquots of the extracted RNA to verify the presence of clear bands and the absence of degradation.

For sequencing the RNA samples, the Oxford Nanopore MK1C sequencer with Flow Cells model R.9.4.1 (Oxford Nanopore Technologies, Oxford, UK) was used. This sequencer has real-time sequencing capability. cDNA was synthesised from the previously extracted RNA using the Direct cDNA Sequencing kit (Oxford Nanopore Technologies). This kit employs a reverse enzyme-based technology for the synthesis of cDNA directly from total RNA. The protocol provided by the manufacturer was followed to generate high-quality cDNA required for sequencing.

Sequencing libraries were prepared with the Ligation Sequencing kit (Oxford Nanopore Technologies, Oxford, UK) in combination with the Native Barcoding Expansion kit (Oxford Nanopore Technologies, Oxford, UK). Two hundred ng of cDNA from each sample was used and the protocols provided for library preparation were followed. A different barcode was assigned to each sample included in the sequencing run, which allowed the multiplexing of the samples and their subsequent identification during the bioinformatics analysis.

### 2.4. Bioinformatics Analysis and Statistics

After sequencing the samples, the reads assigned to each sample were obtained and separated into Transudate, Infectious, and Malignant groups and subjected to analysis using the “*pipeline transcriptome de*” (https://github.com/nanoporetech/pipeline-transcriptome-de, accesed on 16 February 2021), a specific pipeline for the analysis of transcriptomes obtained using Oxford Nanopore’s NGS sequencing technology, which uses snakemake to automate the bioinformatic analysis using minimap2, Salmon [7], edgeR, DEXSeq, and stageR.

This pipeline is used to perform statistical analysis of differential expression and provides tools to identify genes with significant changes in expression between different groups of samples. The reference genome employed for aligning the sequences was homo_sapiens.GRCh38.cdna.all.fa. from Ensemble. Read count normalization was conducted using the Salmon software 0.14.1, with the following parameters: a minimum feature expression of 1, minimum gene and transcription counts of 1, and was only considered genes expressed if in a minimum of 3 samples. The fold change was calculated using the -ddCT method and the differential genes expressed were identified with DESeq2. The False Discovery Rate (FDR) was applied to ensure the significance of each result. For the bioinformatics analysis, a separation of the reads obtained by the Flow Cell was performed assigning the read to its corresponding sample when employing the barcoding strategy to multiplex the sequencing of samples. The sequenced reads were assigned to the clinical groups “Transudate”, “Malignant”, and “Infectious” according to the classification of the corresponding samples. This allowed the comparison of the differences in gene expression between the different groups.

To analyse the expression pattern of each group, samples from the Transudate group were used as normaliser samples for differential expression analysis. The “*pipeline transcriptome de*” was used to identify genes showing significant differences in expression between the Malignant and Infectious groups using the Transudate group as normalization group. Non-parametric tests (Mann–Whitney U test) were performed to determine the significance of the observed differences. Once genes with statistically significant (*p* < 0.05) differential expression were identified, gene ontology pathways and enrichment analysis were performed to better understand the biological functions and metabolic processes associated with these genes. The Human Kegg Orthology database (https://www.genome.jp/kegg/ko.html acceded on 16 February 2021) was used to identify the biological functions enriched in the differentially expressed genes. Enrichment score analysis was performed in order to identify genome-wide expression profiles that show statistically significant, cumulative changes in gene expression that are correlated with a phenotype (malignant or infectious vs. transudate) [8].

Once the genes and biological functions involved in each clinical category were identified, a hierarchical clustering was generated to visualize gene expression patterns between tumour and infectious samples and to find genes with expression patterns statistically significantly distinct from each other [9,10].

## 3. Results

A total of 46 pleural fluids, 11 transudates, 16 infectious, and 19 of malignant origin, were included. The characteristics of the patients included are detailed in Table 1.

### 3.1. Biological Processes Expressed in Infectious Pleural Effusions

**Figure 1:**
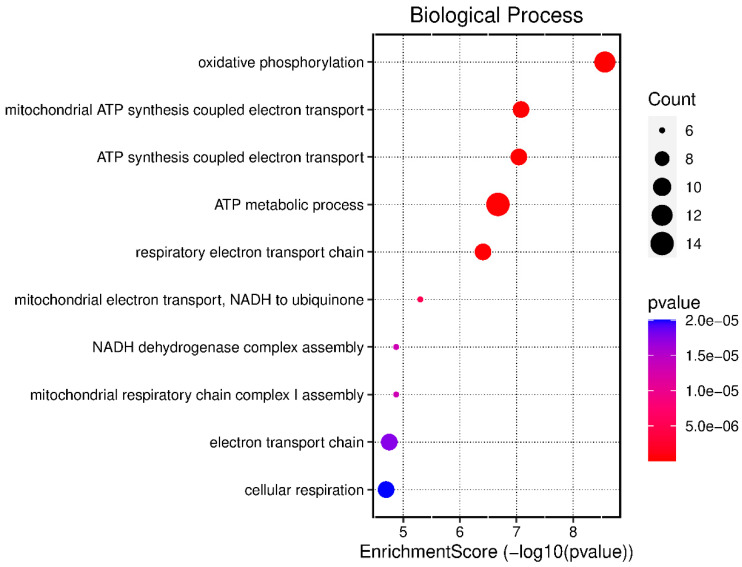
Biological processes expressed in infectious pleural effusions.

Ten biological processes were identified in the samples of infectious pleural effusions that statistically differentiate these fluids from transudates. Biological processes that were identified in the samples of infectious pleural effusions are detailed in Figure 1. In this figure there are represents the enrichment score of these ten processes showing the count (number of detections of an mRNA sequence corresponding to a specific gene) and the *p* value of each of them. The biological processes that showed a higher enrichment score for this diagnostic group were as follows: “oxidative phosphorylation”, with a count of 14 and a *p* value of 5 × 10^−6^, involved in the production of ATP from the energy released during the oxidation of organic compounds; “mitochondrial ATP synthesis coupled electron transport”, with a count of 10 and *p* value of 5 × 10^−6^, which is the process of ATP generation by coupling the electron transport chain and oxidative phosphorylation in the mitochondria; and the biological process “ATP synthesis coupled electron transport” with a count of 8 and *p* value of 5 × 10^−6^, which refers to the process of ATP generation through the coupling of the electron transport chain and oxidative phosphorylation in the cell [11]. The genes involved in the biological processes identified in infectious pleural effusions are listed in Table 2.

### 3.2. Biological Processes Expressed in Malignant Pleural Effusions

**Figure 2:**
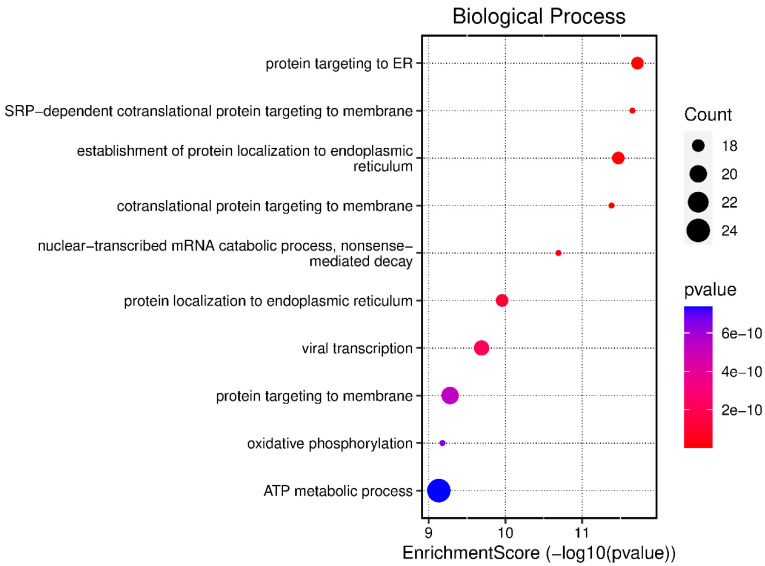
Biological processes expressed in malignant pleural effusions.

The biological processes that showed a higher enrichment score were “protein targeting to the endoplasmic reticulum”, involved in protein synthesis and processing in eukaryotic cells [12], with a count of 18 and *p* value 2 × 10^−10^; “SRP-dependent cotranslational protein targeting to membrane” with a count of 6 and *p* value of 2 × 10^−10^, involved in the action of the signal recognition protein (SRP) complex and its receptor [13]; and “establishment of protein localization to endoplasmic reticulum” with a count of 18 and *p* value of 2 × 10^−10^, related to the process that ensures that proteins are correctly synthesized, folded, and transported to their final destination [14]. All of these results are detailed in Figure 2. The genes involved in the biological processes identified in infectious pleural effusions are listed in Table 3.

### 3.3. Differentially Expressed Genes in Malignant vs. Infectious Effusions

Gene expression in each clinical group, malignant and infectious, normalised with the gene expression in transudates (noninflammatory condition, used as a reference group) was analysed with the Human Kegg Orthology database in order to establish the biological functions involved.

A total of 374 genes were differentially expressed in malignant pleural effusions compared with transudative samples (328 genes downregulated and 46 upregulated), and 176 genes were differentially expressed in infectious exudates compared with transudates (26 downregulated and 150 upregulated).

Table 4 and Table 5 specify the top 20 genes that are overexpressed or repressed in both types of infectious and malignant exudate, respectively.

**Figure 3:**
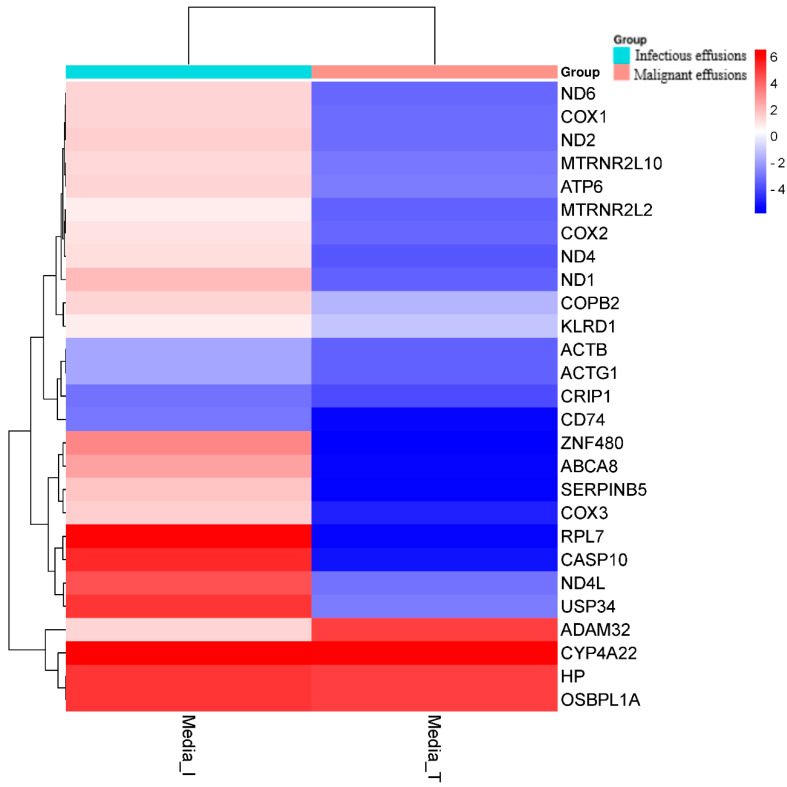
Differentially expressed genes in infectious vs. malignant effusions.

**Figure 4:**
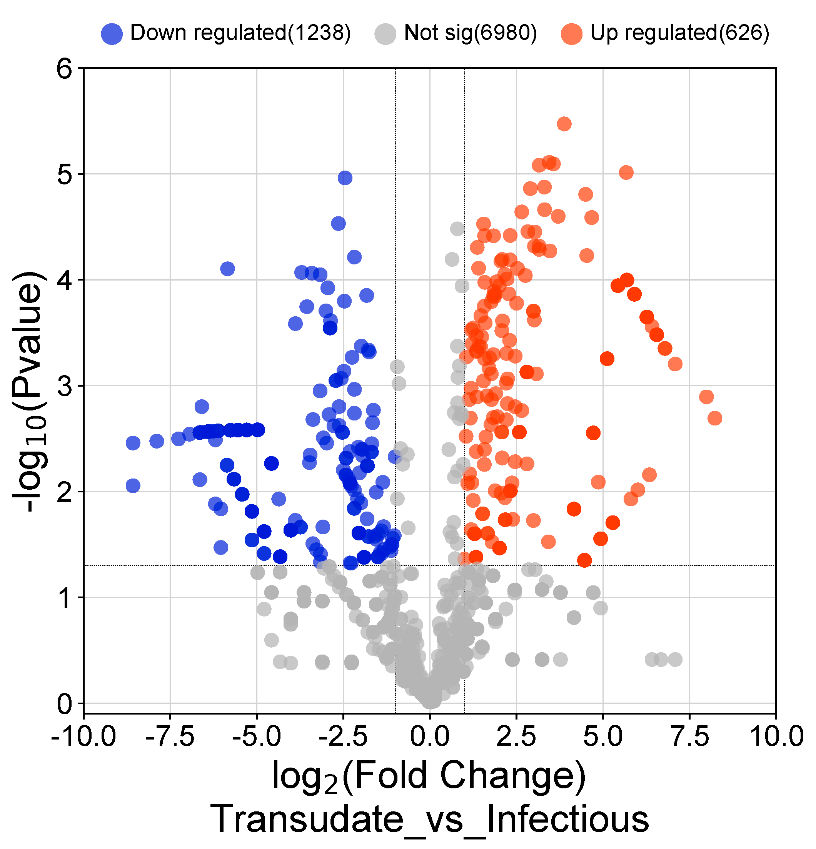
Volcano figure showing the total of overexpressed and repressed genes in infectious vs. transudate pleural effusions.

**Figure 5:**
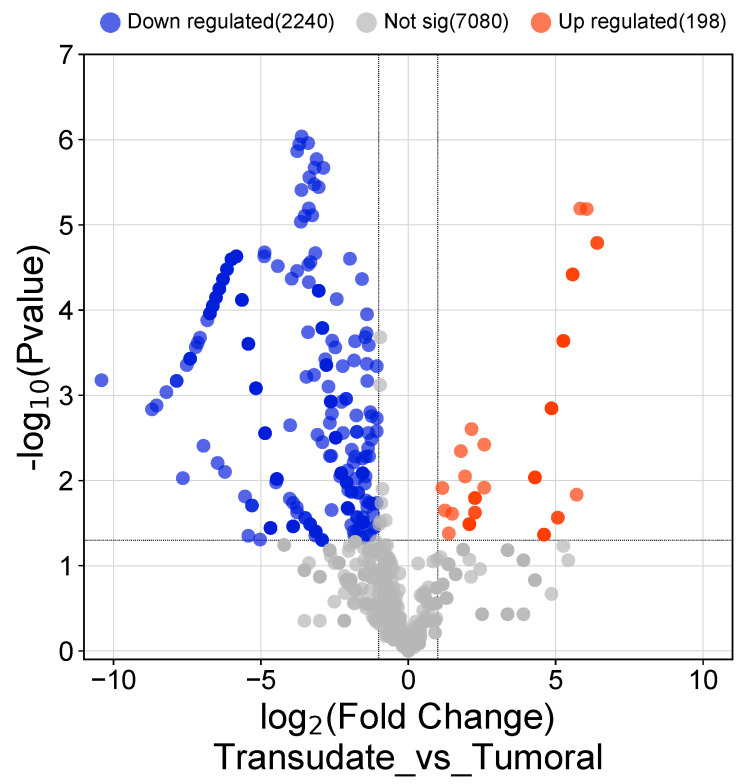
Volcano figure showing the total of overexpressed and repressed genes in malignant vs. transudate pleural effusions.

Twenty-five genes with a statistically significantly different expression patterns were identified when malignant and infectious samples were compared with each other. In the infectious samples, 7 genes were overexpressed compared with the malignant samples, and the identified genes were ZFN80, ABCA8, SERPINB5, RPL7, CASP10, ND4L, and USP34. In the malignant samples compared with the infectious samples, only the gene ADAM32 was overexpressed. The different biological processes expressed in malignant and infectious effusions are separately detailed (Figure 3). Figure 4 and Figure 5 shows the total number of overexpressed and repressed genes in infectious versus transudate pleural effusions and in tumoral versus transudate pleural effusions respectively as a volcano figure.

## 4. Discussion

Recent breakthroughs in transcriptome analysis have allowed us to describe two different findings: first, the discovery of differentially expressed genes in malignant vs. infectious effusions, with all the potential applications that this implies, and secondly, to define the different biological processes involved in the inflammatory mechanisms of malignant and infectious pleural effusions.

The comparative analysis of gene expression enables us to identify the expression profiles and to highlight those genes that showed distinct expression patterns between the groups; this could indicate their potential as biomarker candidates to discriminate between malignant and infectious aetiologies or biomarkers with prognostic capabilities.

The mRNA of specific conventional biomarker genes such as Lung-specific X (LUNX) and vascular endothelial growth factor (VEGF) has been evaluated to distinguish between MPEs and benign pleural effusions [15]. More recently, the mRNA of combinations of four specific biomarker genes showed promising results in the diagnostic classification and prognosis of malignant pleural effusions [16]. Our contribution is a more comprehensive study that has included all mRNA and, through bioinformatics analysis, has established which genes were differentially expressed in malignant and infectious effusions.

The main objective of the study has been to contribute to a first approximation of the knowledge of the genes involved, and it has not been designed for the purpose of establishing diagnoses with these biomarkers. Nevertheless, the findings allow us to suggest lines for future research on evident clinical utility.

A few examples of comparisons of gene expression may contribute to understanding its potential. For instance, we have found that the ADAM (A Disintegrin and Metalloproteinase) gene is overexpressed in malignant pleural effusions. A few investigations report that the expression of several ADAM is upregulated in cancer cells [17], including lung cancer [18]. This allows a consistent interpretation that they could prove to be useful biomarkers in malignant effusions or even suggest therapeutic targets.

Similarly, different expression in several genes has been found to be associated with infectious pleural effusions. In this first approach, probably the ones that could be most noteworthy are ZFN480, ABCA8, SERPIN, ribosomal protein L7 (RBL7), CASP10 ND4L, and USP34 [19,20,21,22,23]. All these genes and their derived proteins have shown an important relationship with the inflammatory processes associated with infection, in their production or in the host response.

As genes can be differently expressed in malignant and infectious pleural effusions, the detection of their presence, absence, or degree of activity could represent potential biomarker combinations in pleural effusion.

Based on our knowledge of gene expression, we have been able to explore the mechanisms involved in the production or persistence of pleural effusions, establishing their biological processes with the network analysis using the Human Kegg Orthology database, which allows pathway identification [24].

A biological process in transcriptomics refers to the series of molecular events occurring within a particular cell or organism that carries out a specific biological function and can be inferred from the identification of genes that are coordinately regulated and involved in that biological function [25]. For example, if genes that act in a coordinated manner in the immune response are identified, it is consequently inferred that the biological process that is occurring is the immune response.

Our study provides new information on the biological processes associated with malignant and infectious pleural effusions, using a very ambitious method that analyses (through mRNA) the processes that are activated (producing proteins) in that situation. This differentiates it from the study of proteins that can be found without being an active reflection of a disease or dysfunction. This type of approach has been used in the investigation of some diseases but has not been applied to the study of pleural effusions [25].

In our study, we have been able to identify the involvement of clearly differentiated biological processes in malignant and infectious effusions. In malignant effusions, the transcriptomics analysis reveals the expression of biological processes mainly associated with protein synthesis and transport. This finding is consistent with the situation occurring in a carcinogenic context, where protein and cell membrane synthesis may be altered due to mutations in genes related to these processes. In addition, cancer cells often have different metabolic needs from normal cells and may require altered protein and cell membrane synthesis to maintain their growth and survival [26].

On the other side, in infectious effusions, the detected biological processes were those involved in metabolic processes affecting ATP production, cellular respiration, and the electron transport chain. These are well-known phenomena in the presence of bacterial infection, since bacteria use components of the host’s internal environment to produce ATP through cellular respiration [27].

Knowledge and understanding of the biological processes involved in the development and progression of pleural effusions are an opportunity for the development of diagnostic strategies for searching for prognostic biomarkers and for the orientation of new therapeutic targets.

Although our study represents a new approach to the knowledge and study of pleural diseases, it has some limitations that should be considered. Transcriptome study technologies are currently complex and relatively expensive, although rapid development may facilitate their use. It is also important to note that once the genes involved in biological processes have been detected, their detection by other standard molecular methods is possible, which would allow their clinical application. In this descriptive exploratory study, validation with qPCR has not been carried out, which is a limitation.

This study, moreover, was carried out in a single centre and with a relatively small number of patients due to the logical limitations imposed by the technical complexity. However, the pathology of the patients was well defined and is reasonably applicable to other clinical settings. Furthermore, the results obtained are consistent with the scientific knowledge available for malignant and infectious diseases.

The methodology employed in this study opens a new field of opportunities for the study of the mechanisms involved in pleural diseases. The detection of gene expression (by means of mRNA) makes it possible to detect the active functions in a particular process.

## 5. Conclusions

The conclusions of this work are focused on two aspects. A first approach has been made to the comparison of gene expression in these effusions. Again, different genes with obvious potential for future research have been identified. It is reasonable to assume that the study of these genes or their various combinations may provide diagnostic or prognostic information in patients with pleural effusions. In addition, biological processes associated with malignant and infectious pleural effusions have been identified and are clearly distinct, improving our understanding and knowledge of the mechanisms of pleural disease.

## Figures and Tables

**Table 1:** Demographics and characteristics of the study group.

CHRACTERISTICS	Transudates (11)	Infectious (16)	Malignant (19)
Male, n(%)	3 (27.27%)	9 (56.25%)	11 (57.89%)
Age, year	79.18 ± 11.6	63.38 ± 12.3	65.89 ± 13.9
DISEASES	Congestive heart failure (7)	Paraneumonic (13)	Lung cáncer (6)
	Another transudate causes (4)	Another infectious (3)	Breast cancer (4)
			Another cáncer (9)

**Table 2:** Genes involved in the biological processes identified in infectious pleural effusions.

ID	Description	*p* Value	Gene ID
GO:0006119	oxidative phosphorylation	2.75522 × 10^−9^	ATP8/ND4L/ND1/COX3/PPIF/ND4/ND6/COX1/ATP6/ND3/COX2/ND2
GO:0042775	mitochondrial ATP synthesis coupled electron transport	8.32541 × 10^−8^	ND4L/ND1/COX3/ND4/ND6/COX1/ND3/COX2/ND2
GO:0042773	ATP synthesis coupled electron transport	9.10531 × 10^−8^	ND4L/ND1/COX3/ND4/ND6/COX1/ND3/COX2/ND2
GO:0046034	ATP metabolic process	2.1265 × 10^−7^	ATP8/ND4L/ND1/GAPDH/COX3/PPIF/ND4/ND6/COX1/ATP6/ND3/COX2/AK9/ND2
GO:0022904	respiratory electron transport chain	3.90402 × 10^−7^	ND4L/ND1/COX3/ND4/ND6/COX1/ND3/COX2/ND2
GO:0006120	mitochondrial electron transport, NADH to ubiquinone	4.9925 × 10^−6^	ND4L/ND1/ND4/ND6/ND3/ND2
GO:0010257	NADH dehydrogenase complex assembly	1.33194× 10^−5^	ND1/ND4/ND6/ND3/NDUFAF8/ND2
GO:0032981	mitochondrial respiratory chain complex I assembly	1.33194 × 10^−5^	ND1/ND4/ND6/ND3/NDUFAF8/ND2
GO:0022900	electron transport chain	1.76432 × 10^−5^	ND4L/ND1/COX3/ND4/ND6/COX1/ND3/COX2/ND2
GO:0045333	cellular respiration	2.00624 × 10^−5^	ND4L/ND1/COX3/ND4/ND6/COX1/ND3/COX2/ND2

**Table 3:** Genes involved in the biological processes identified in malignant pleural effusions.

ID	Description	*p* Value	Gene ID
O:0045047	protein targeting to ER	1.90903 × 10^−12^	RPL8/RPL36/RPS6/RPL32/RPL30/RPL15/ RPL6/RPL13/RPS20/RPS12/RPL7/RPS14/ RPLP2/RPL28/SEC63/HSPA5/RPL37/RPL29
GO:0006614	SRP-dependent cotranslational protein targeting to membrane	2.21935 × 10^−12^	RPL8/RPL36/RPS6/RPL32/RPL30/RPL15/ RPL6/RPL13/RPS20/RPS12/RPL7/RPS14/ RPLP2/RPL28/SEC63/RPL37/RPL29
GO:0072599	establishment of protein localization to endoplasmic reticulum	3.39106 × 10^−12^	RPL8/RPL36/RPS6/RPL32/RPL30/RPL15/ RPL6/RPL13/RPS20/RPS12/RPL7/RPS14/ RPLP2/RPL28/SEC63/HSPA5/RPL37/RPL29
GO:0006613	cotranslational protein targeting to membrane	4.15652 × 10^−12^	RPL8/RPL36/RPS6/RPL32/RPL30/ RPL15/RPL6/RPL13/RPS20/RPS12/RPL7/ RPS14/RPLP2/RPL28/SEC63/RPL37/RPL29
GO:0000184	nuclear-transcribed mRNA catabolic process, nonsense-mediated decay	2.04422 × 10^−11^	RPL8/RPL36/RPS6/RPL32/RPL30/ SMG1/RPL15/RPL6/RPL13/RPS20/ RPS12/RPL7/RPS14/RPLP2/RPL28/RPL37/RPL29
GO:0070972	protein localization to endoplasmic reticulum	1.10638 × 10^−10^	RPL8/RPL36/RPS6/RPL32/RPL30/ RPL15/RPL6/RPL13/RPS20/RPS12/RPL7/ RPS14/RPLP2/RPL28/SEC63/HSPA5/RPL37/RPL29
GO:0019083	viral transcription	2.04724 × 10^−10^	NELFB/RPL8/RPL36/RPS6/RPL32/ RPL30/RPL15/RPL6/RPL13/RPS20/RPS12 RPL7/RPS14/RPLP2/POM121C/RPL28/ TARDBP/RPL37/RPL29
GO:0006612	protein targeting to membrane	5.26086 × 10^−10^	RPL8/RPL36/RPS6/RPL32/RPL30/KCNB1/ RPL15/RPL6/RPL13/RPS20/RPS12/RPL7/ RPS14/RPLP2/RPL28/SEC63/HSPA5/ RPL37/RPL29/ITGB2
GO:0006119	oxidative phosphorylation	6.61515 × 10^−10^	COX1/ND4/ND6/COX3/COX2/ND1/ PINK1/ATP5PD/ND5/ND4L/CYTB/ SDHC/ATP6/ND2/VCP/PDE12/FXN
GO:0046034	ATP metabolic process	7.36436 × 10^−10^	COX1/ND4/ND6/COX3/COX2/ND1/ SLC4A1/PINK1/ATP5PD/PFKFB4/ ND5/ND4L/HSPA8/CYTB/PKM/SDHC/ POM121C/ATP6/ND2/ENO1/ALDOA/ VCP/PDE12/FXN

**Table 4:** List of the top 20 upregulated and top 20 downregulated molecules in the infectious pleural effusion.

**List of the Top 20 Up-Regulated Molecules in the Infectious Pleural Effussion**			
**Gene Symbol**	**Gene Name**	**logFC**	***p*-Value**
UBE2S	Ubiquitin Conjugating Enzyme E2S	7.08513297	0.03788703
ST6GALNAC5	ST6 N-Acetylgalactosaminide Alpha-2,6-Sialyltransferase 5	6.26329843	0.0257876
SMAD4	Mothers against decapentaplegic homolog 4	6.26329843	0.0257876
RPL7A	Ribosomal Protein L7a	6.26329843	0.0257876
RGSL1	(Regulator Of G Protein Signaling Like 1	6.54938616	0.03166481
PGBD1	PiggyBac Transposable Element-Derived 1	6.54938616	0.03166481
PARP15	Poly(ADP-Ribose) Polymerase Family Member 15	5.90608458	0.01969065
NLGN4Y	Neuroligin 4 Y-linked	5.90608458	0.01969065
LPAL2	Lipoprotein(a) like 2 (pseudogene)	6.78802257	0.03396501
LOC100419170	Toll like receptor 2 pseudogene	6.26329843	0.0257876
L3MBTL3	Lethal(3)malignant brain tumor-like protein 3	5.68769633	0.01856111
FRA10AC1	FRA10A Associated CGG Repeat 1	6.54938616	0.03166481
DNAJC10	DnaJ Heat Shock Protein Family (Hsp40) Member C10	6.78802257	0.03396501
DGKE	Diacylglycerol Kinase Epsilon,	5.68769633	0.01856111
DDB1	Damage Specific DNA Binding Protein 1	5.68769633	0.01856111
CYP4A22	Cytochrome P450 Family 4 Subfamily A Member 22	6.54938616	0.03166481
CMC1	COX assembly mitochondrial protein 1 homolog	5.90608458	0.01969065
CCNC	Cyclin-C	5.90608458	0.01969065
ATP10B	ATPase Phospholipid Transporting 10B	6.41342214	0.02907238
ARF4	ADP Ribosylation Factor 4	5.68769633	0.01856111
**List of the Top 20 Down-Regulated Molecules in the Infectious Pleural Effussion**			
**Gene Symbol**	**Gene Name**	**logFC**	***p*-Value**
NCL	Lipofuscionosis neuronal ceroidea	−5.84529934	0.01856111
C15orf40	Chromosome 15 opne reding frame 40	−3.88554066	0.027804565
DCTN3	Dynactin subunit 3 protein	−3.70467213	0.01856111
RPL10	Ribosomal Protein L10	−3.55664134	0.023162056
HADHA	Hydroxyacil CoA dehydrogenase trifunctional mutienzyme complex subunit alpha	−3.40781869	0.01856111
PPFIA1	Protein Tyrosine Phosphatase receptor type F polypeptide	−3.16943063	0.01856111
CRIP1	Cysteine-Rich protein 1 human recombinant	−3.00284513	0.02430739
CTSB	Cathepsin B	−2.95194295	0.019054027
CD74	Cluster of differentiation 74	−2.88370022	0.029072383
LEPROTL1	Leptin receptor overlapping transcript like 1	−2.88370022	0.029072383
PPIF	Peptidylprolyl Isomerase F	−2.88370022	0.029072383
MX2	MX dynamin like GTPase 2	−2.87009164	0.027066857
ALOX5AP	Arachidonate 5-lipoxygenase activating protein	−2.71636567	0.049560165
POM121L15P	POM121 transmembrane nucleoporin like 15, pseudogen	−2.71636567	0.049560165
RNF213	Ring fing protein 213	−2.64251631	0.017582598
RPL4	Large ribosomal subunit protein L4	−2.56186959	0.048122461
KPNB1	Karyopherin subunit beta 1	−2.48646613	0.043130785
GAPDH	Glyceraldeyde-3-phosphatase deshydrogenase	−2.47163268	0.021680223
PCSK5	Proprotein convertase subtilisin/kexin type 5	−2.45241044	0.015410669
CORO1C	Coronin 1c	−2.24808151	0.033965007

**Table 5:** List of the top 20 upregulated and top 20 downregulated molecules in the malignant pleural effusion.

**List of the Top 20 Up-Regulated Molecules in the Tumoral Pleural Effussion**			
**Gene Symbol**	**Gene Name**	**logFC**	***p*-Value**
CYP4A22	Cytochrome P450 family 4 subfamily A member 22	6.410457063	0.005199019
NELFB	Negative elongation factor complex member B	6.410457063	0.005199019
ESCO1	Establishment of sister chromatid cohesion N-acetyltransferase 1	6.052724677	0.004716469
ZCCHC24	Zinc finger CCHC-type containing 24	5.833949909	0.004716469
CYB5R3	Cytochrome b5 reductase 3	5.575965026	0.006286137
PFKFB4	6-phosphofructo-2-kinase/fructose-2,6-biphosphatase 4	5.575965026	0.006286137
ZNF43	Zinc finger protein 43	5.575965026	0.006286137
RIN2	Ras and Rab interactor 2	5.261578232	0.012908359
GRM2	Glutamate metabotropic receptor 2	5.261578232	0.012908359
WDR72	WD repeat domain 72	5.261578232	0.012908359
SLC25A30	Solute carrier family 25 member 30	5.261578232	0.012908359
PTPN20	Protein tyrosine phosphatase non-receptor type 20	5.261578232	0.012908359
HLA-C	Major histocompatibility complex, class I, C	5.261578232	0.012908359
SULT1C2P1	Sulfotransferase Family 1C	4.859022623	0.041574119
HP	Haptoglobine	4.859022623	0.041574119
AGAP12P	ArfGAP GTPase, ankyrin repeat PH domain 12, pseudogene	4.859022623	0.041574119
SSX4B	SSX family member 4B	4.859022623	0.041574119
LOC642929	General Transcription Factor II, I Pseudogen	4.859022623	0.041574119
**List of the Top 20 Down-Regulated Molecules in the Tumoral Pleural Effussion**			
**Gene Symbol**	**Gene Name**	**logFC**	***p*-Value**
SAMD12	Sterile Alpha Motif Domain Containing 12	−10.40752199	0.027309577
C1orf116	Chormosome 1 Open Reading Frame 116	−8.692680487	0.042610742
FANCI	Fanconi Anemia Complementary group 1	−8.538352536	0.041574119
NASP	Nuclear Autoantigenic Sperm Protein	−8.203765305	0.030086669
GLIPR1L2	GLI pathogenesis-related 1 like 2	−7.8571759	0.027309577
COL28A1	Collagen Type XXVIII Alpha 1 Chain	−7.8571759	0.027309577
CLU	Clustering	−7.514895677	0.021227484
FOXN3	Forkhead Box N3	−7.400075641	0.018726604
AGBL5	Cytosolic carboxypeptidase-like protein 5	−7.400075641	0.018726604
PABIR3	PABIR family number 3	−7.400075641	0.018726604
ANKS6	Ankyrin Repeat And Sterile Alpha Motif Domain Containing 6	−7.208650249	0.013964234
CARS1	Cysteinyl-TRNA Syntethase 1	−7.138748507	0.012908359
TRIM73	Tripartite Motif Containing 73	−7.065286645	0.012908359
TECPR2	Tectonin Beta-Propeller Repeat Containing 2	−6.819377539	0.010672183
PSMD11	Proteasome 26S Subunit, Not-ATPase 11	−6.727119503	0.008987272
SMAD6	Supressor of Mothers against Decaapentaplegic 6	−6.727119503	0.008987272
SCML4	Scm Polycomb GroupnProtein Like 4	−6.727119503	0.008987272
MFSD14CP	Major Facilitator Superfamjily Domain Containing 14C	−6.727119503	0.008987272
GBP4	Guanilate Binding Protein 4	−6.628556208	0.007560163
XKR9	XK erlated protein 9	−6.628556208	0.007560163

## Data Availability

Data are contained within the article.
